# Predicting Pancreas Cell Fate Decisions and Reprogramming with a Hierarchical Multi-Attractor Model

**DOI:** 10.1371/journal.pone.0014752

**Published:** 2011-03-14

**Authors:** Joseph Xu Zhou, Lutz Brusch, Sui Huang

**Affiliations:** 1 Center for Information Services and High Performance Computing, Technical University Dresden, Dresden, Germany; 2 Institute of Biocomplexity and Informatics, University of Calgary, Calgary, Canada; 3 Kavli Institute for Theoretical Physics, University of California Santa Barbara, Santa Barbara, California, United States of America; Keio University, Japan

## Abstract

Cell fate reprogramming, such as the generation of insulin-producing β cells from other pancreas cells, can be achieved by external modulation of key transcription factors. However, the known gene regulatory interactions that form a complex network with multiple feedback loops make it increasingly difficult to design the cell reprogramming scheme because the linear regulatory pathways as schemes of causal influences upon cell lineages are inadequate for predicting the effect of transcriptional perturbation. However, sufficient information on regulatory networks is usually not available for detailed formal models. Here we demonstrate that by using the qualitatively described regulatory interactions as the basis for a coarse-grained dynamical ODE (ordinary differential equation) based model, it is possible to recapitulate the observed attractors of the exocrine and β, δ, α endocrine cells and to predict which gene perturbation can result in desired lineage reprogramming. Our model indicates that the constraints imposed by the incompletely elucidated regulatory network architecture suffice to build a predictive model for making informed decisions in choosing the set of transcription factors that need to be modulated for fate reprogramming.

## Introduction

A gene regulatory network (GRN) in which fate-determining transcription factors (TFs) regulate each other drives the development of tissues by orchestrating the activation or suppression of the appropriate genes across the genome to establish the steady-state gene expression patterns that specify a given cell type [1]. Ever since the recognition of gene regulation it has been proposed that cell differentiation into a variety of cell types is due to the emergence of multiple stable attractor states in GRNs which guarantee the stability of the cell type specific expression patterns [2], [3], [4]. The recent integrated analysis of gene expression profiles have provided evidences that cell types represent attractor states of the dynamics of GRNs [3], [5], [6]. If the cell-type specific genomic expression configurations are attractors, then they are “pre-programmed” by the particular wiring diagram (architecture) of the GRNs. Accordingly, because of this self-organizing property of entire gene expression patterns that are commensurate for a particular cell fate, the activation of one or a few key “fate determining TFs” suffices to switch cell lineages (transdifferentiation) [3], [7].

Although early transdifferentiation experiments or reprogramming between related cell lineages revealed this expected cell line plasticity and self-organization [7], [8], [9] they have received little attention because of the deeply rooted dogma of immutability between cell lineages. Such reprogrammability has seen a revival in the past years owning to the increasing understanding of some governing principles of fate determination by the transcriptional network and the recent interest in the successful reprogramming of cell phenotypes for regenerative medicine, including the conversion of a variety of adult somatic cells into the embryonic stem cell like state [10].

Lineage reprogramming reinforces the notion that the determinant of lineage identity is embodied in the dynamics of regulatory networks rather than simply in the pattern of static “epigenetic” chromatin marks, represented by covalent histone and DNA modifications [3], [11]. The picture is emerging that these covalent epigenetic marks act as local gene activity switches whereas the transcription factors are the prime regulator of specific gene expression patterns because they form networks which are naturally necessary to coordinate the expression between the gene loci across the genome [12]. The covalent epigenetic marks may play only secondary role, perhaps by providing additional discrimination of expression status between individual genes because the enzymatic apparatus which modifies the DNA and histones lack gene locus specificity and are reversible anyway [13]. Thus, it is not surprising that reprogramming can be achieved by controlling TF expression without bothering with covalent modifications of DNA or chromatin.

Recent successes in reprogramming cells for regenerative medicine purposes via ectopic TFs have been achieved largely by educated guess about which TFs needs to be over-expressed combined with systematic, brute-force trial and error ectopic expression of combinatorial sets of relevant TFs (see below). A typical, first-order rationale is that the TF normally expressed in the desired target lineage (lineage-specific TFs) may also serve as lever for reprogramming a cell to that lineage and in fact, this has been demonstrated for numerous cases. However, given the nonlinear dynamics of GRNs, the assumption of such linear relationship between cell state and TF expression, which also interprets correlation as causation, is simplistic. For instance, many key regulators need only be active transiently to achieve permanent reprogramming [7].

As information on the GRN wiring diagrams is rapidly accumulating (albeit far from complete), the time is ripe to ask whether the optimal reprogramming strategy can be predicted based on our knowledge of the incomplete but increasingly complex GRN architecture that are being reported. The complexity of the regulatory network with feedback loops and cross-talks suggests that a formal mathematical modeling that integrates the actions of interacting TFs into the network dynamics will outperform existing empirical approaches based on qualitative, linear and *ad hoc* hypotheses.

Here we set out to demonstrate how the development of cell lineages in the pancreas can be described using a simple mathematical model based on rate equations that capture the mutual influences of TF expression reported in the literature. We use a qualitative but formal modeling paradigm to model the development of the major cell lineages of the pancreas: the exocrine cells and the endocrine cells, including β, δ and α islet cells from the common Pdx1-positive precursor cells. Using a system of elementary nonlinear rate equations to describe the mutual regulatory influence of ten TFs involved in the pancreas development, we present a minimal model that qualitatively captures known interactions and is able (i) to recapitulate the robust generation of the various cell lineages of the pancreas as defined by gene expression patterns; (ii) to predict the temporal changes of key TFs in the development of particular cell lineages; (iii) to predict the outcomes of gene knock-outs; (iv) to predict the outcome and to help to design new recipes of reprogramming experiments. Our modeling approach thus represents a first step beyond the qualitative interpretation of linear pathways when the paucity of information precludes more detailed modeling.

## Results

### A pragmatic modeling paradigm for incomplete data

The incomplete and often circumstantial and ambiguous information on regulatory interactions preclude modeling in the traditional sense, as employed in engineering, in which one aims at a maximally detailed model description with measured or fitted quantitative parameters. Our goal is not to truthfully incorporate all known interactions into a complete model and then predict testable behaviours in response to perturbations of a (presumably) well-determined system. In contrast, we ask whether given the available information which is complex enough to preclude the simple hand-waving type of argumentation, yet too incomplete for a comprehensive model, any formal but minimal modeling approach can offer insights on the collective function of fate determining TFs.

In other words, we seek to answer the following pragmatic question: does the qualitative information on functional and regulatory relationships between key TFs reported in the literature generate sufficient constraints on the dynamics of a system so that one can formalize how cell fate commitment, natural or ‘reprogrammed’, emerges from the dynamics of a GRN? More specifically, does network topology alone without knowledge of the quantitative nature of interactions (real values of rate coefficients) suffice to formally predict the dynamical behaviour of the network? Studies of simple parameter-free systems, such as discrete Boolean networks, suggest that the characteristic global dynamics of networks, including the presence of stable attractors, do not depend much on quantitative details of interaction parameters but rather, on the network architecture [14].

**Figure 1:**
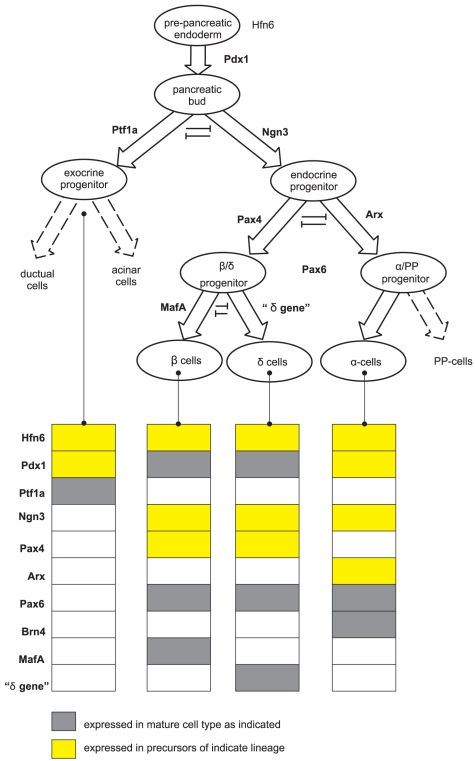
Cell lineages of pancreatic cell differentiation and their gene expression patterns. Mouse pancreas development starts from the *Pdx1* ^+^ cells, which gradually differentiate into exocrine, α, β and δ cells. Genes marked with yellow color are transiently expressed while those with grey color are permanently expressed in mature cells.

To study how the known set of regulatory interactions collectively lead to a dynamics in which the attractor states correspond to observable cell types or lineages, we focused on the fate options of the multipotent pancreas precursor cells that express the TF Pdx1 [15], [16], [17], [18], [19]. We asked how they commit, via a three levels hierarchy of binary cell fate decisions into various lineages (Fig. 1): (i) At the first level, the branching into the *exocrine* and *endocrine* lineages; (ii) at the second level, the fate decision of the Ngn3 positive endocrine progenitor cell between either the and β / δ or the α (glucagon producing) lineages; and (iii) at the third branching, where the β / δ precursors commit to produce either β cells or δ cells, respectively.

Such a hierarchical sequence of mostly binary cell fate decisions appears to be a universal mechanism through which higher metazoan produce the diversity of cell types and has been well studied in hematopoiesis, neurogenesis and in early embryo development [14], [20], [21], [22], [23].

Since the characteristic cell fate dynamics with binary branch points and discrete stable states must somehow emanate from the dynamics of the GRN, the operational question is whether the same qualitative knowledge used in ad hoc argumentation by experimentalists can, when formalized as a simple dynamical system using a set of ODEs, achieve predictive capability beyond the existing qualitative argumentation. Moreover, we also would like to explore the superiority of a formal model in predicting the artificial switching of cell fates by genetic manipulation of fate-determining TFs. Current reprogramming methods rely on *ad hoc* arguments and brute-force trial and error experiments to identify the relevant genetic lever points in the network whose manipulation can achieve cell fate switching. For instance, systematic combinatorial screening led to the identification of a set of regulators, Ngn3, Pdx1 and MafA, which are over-expressed jointly to convert exocrine cells to β-like cell [24]. Conversely, based on the qualitative information on the role of Pax4 in the decision between α and the β / δ cells it was found that ectopic expression of Pax4 targeted to pancreas progenitor or α-cells converts these cells to β cells [25].

### Pancreas cell fate regulation

**Figure 2:**
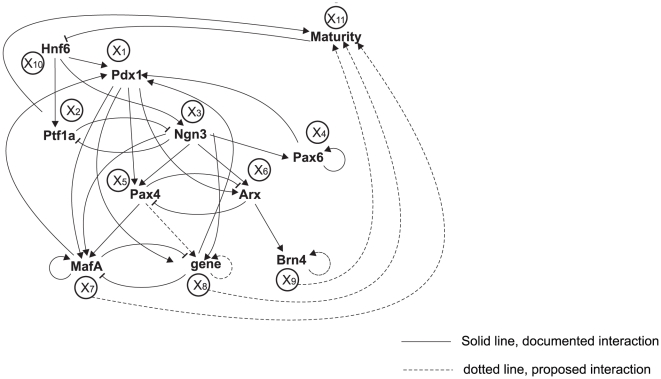
Gene regulatory network for pancreatic cell differentiation. Master model: *Hnf6* activates *Pdx1*, *Ptf1a* and *Ngn3*. Three cross-inhibition gene pairs are *Ptf1a-Ngn3, Pax4-Arx* and *MafA-δ gene*. Nodes are denoted by TF names. Arrow-heads denote activation while flat-heads denote inhibition. Circles are variable names in the mathematical model. An alternative model: Pdx1 directly inhibits both Ptf1a and Ngn3.

In the pancreas, the pairs of opposing TFs that control binary decisions at the three levels of interest here have been identified and to some extent their regulatory properties characterized (see [16], [26], [27], [28] for review). The first level of branching, between exocrine and endocrine pancreas, is governed by the mutually suppressing pair of the TFs, Ptf1a <--> Ngn3 [17] (Fig. 2). Thus, Pt1a and Ngn3 are the fate specific markers of exocrine and endocrine progenitor cells, respectively. They also are sufficient and necessary for the development and function of the exocrine and endocrine pancreas, respectively. The second-level branching is governed in a similar manner by the Pax4 <--> Arx circuit which determines the β / δ vs. the α-cell lineage, respectively [16]. The fate-determining TFs for the third branching into the β-cells vs. the δ cells have not fully been characterized. However, TFs have been shown to bias the decision to and be necessary for establishing the β-cells, notably MafA [16] whereas little information is available for fate determining factor for the δ cells. Thus, for modeling purposes and for maintaining symmetry, we use a place-holder for the δ -cell determining factor, called “δ factor” and then assume a third pair of opposing TFs: MafA <- -> δ factor, governing the determination of β vs. δ cells, respectively (Fig. 2).

At this point a key question arises: how are these binary switch circuits which readily explain the binary nature of the respective decision by progenitors, integrate into a single network that orchestrates the hierarchical development of pancreas progenitor to the various cell lineages by activating the correct fate decision in the appropriate cells (Fig. 1)? Little is known about the integrated behaviour of a system consisting of a linked set of toggle switches or similar circuits [29], [30]. A simple generic model for how such coupling in principle can generate a hierarchically branching system has been proposed in which execution of higher-level decisions shift the lower-level decision points into the regime of bistability such that the undecided cell is placed in the unstable state of indeterminacy between the two attractors, hence driving the next level binary decision [31]. Here we propose a similar model for a more complex system which, however, is informed by the specific knowledge of a selected set of regulatory relationships.

Because our model will not predict the precise ratio of the different cell types during each differentiation due to lack of pertinent information, the cellular signal transduction pathways which control and fine-tune the fate decisions in response to extracellular cues are not included in the model. Instead, the model captures largely the intrinsic unfolding of the multiple lineages coordinated by the transcriptional regulatory interactions between the fate controlling TFs. However, to ensure the arrow of time (directionality) of development which can result from deterministic or stochastic influences [32], we also incorporated (i) a deterministic extrinsic factor “maturation” that signals the approaching of terminal differentiation of the tissue (see **section:** ***Methods***) and (ii) a stochastic process in each equation that accounts for the stochasticity of TF expression and cell fate commitment [28], [33].

### Basic simulation results: cell type diversification

#### Prediction of lineages/cell types and their expression profiles

To simulate the cell development dynamics of the above GRN model above, we choose the initial conditions of the expression level of all the pancreatic genes to be zero for all genes except for gene *Hnf6* and *Pdx1* ***.*** We run our single cell gene network model in a cell population. Each cell in the population has the same network model and the same initial conditions with high expression of *Hnf6* and *Pdx1*. Due to the stochastic gene expression terms in the model; our GRN model produces all steady-states (attractors) of gene expression patterns of four cell types and their gene expression time profiles during the development. The temporal behaviours during pancreas development of some for the genes of the network have been described and summarized in the literature [34]. These time courses will, in addition to producing the distinct cell types, serve for qualitative validation for our model.

**Figure 3:**
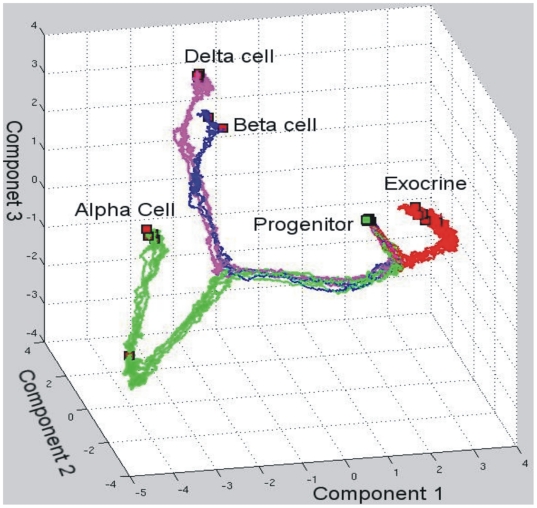
Three branchings of gene expression profiles during pancreatic cell differentiation. This figure describes the dynamics of three bifurcations happened between three cross-inhibition gene switches: *(A) Ptf1a - Ngn3, (B) Pax4 - Arx (C) MafA - δ cell gene*. The left panels show gene expression profiles. The right panels show phase diagrams of the cross-inhibition genes.

Fig. 3 presents three “branchings” of gene expression trajectories during the pancreas cell differentiation. The expression level of *Hnf6* starts at a high level and gradually decays as observed in experiments [34]. It activates the first switch between Ptf1a and Ngn3. As shown in Fig. 3A, the non-deterministic property allows the state trajectory to split, in this case, into two cell lineages that either express high Ptf1a and low Ngn3, which corresponds to the exocrine linage, or vice versa, which corresponds to the endocrine progenitors. The choice of either trajectory at this bifurcation point is stochastic. Once a cell passes the branching point, the two genes are regulated in opposite directions according to the bistable circuit. If cells follow the endocrine linage, they become Ngn3 positive which dominates over Ptf1a (“lower branching lines” in Fig. 3A, right panel). High Ngn3 triggers the second switch, embodied by the branching governed by Pax4 and Arx (Fig. 3B). Later Ngn3 decreases, reflecting its observed transient expression character, as Hnf6 is down-regulated with maturation (see below). Endocrine progenitors at this stage can differentiate into either α cells or β/δ cell progenitors. Cells fated to the trajectory with high Arx (and low Pax4) turn on the Arx target gene Brn4 which is a marker gene for the α phenotype and whose expression persists, as observed, even if the lineage determining factor Arx decreases (Fig. 3B). By contrast, in cells fated to the trajectory with dominating Pax4, this TF then triggers the last switch that controls the branching between MafA and the δ cell gene (Fig. 3C) which subsequently will activate the respective effector genes, producing the distinct β and δ cells.

The first two bistable switches (Ptf1a-Ngn3 and Pax4-Arx) belong to the type of supercritical pitchfork bifurcation which makes the transition from a mono-stable (pre-decision) to the bi-stable (post-decision) regime. In such decision points, the activities of the switch genes in the mono-stable regime go to zero if the upstream input signal vanishes. In contrast, the third switch, MafA-δ gene, is modelled to exhibit a type of bifurcation which makes the transition from a tri-stable (pre-decision) to a bi-stable (post-decision) regime. Here, the switch genes maintain their values even if the upstream signal vanishes.

**Figure 4:**
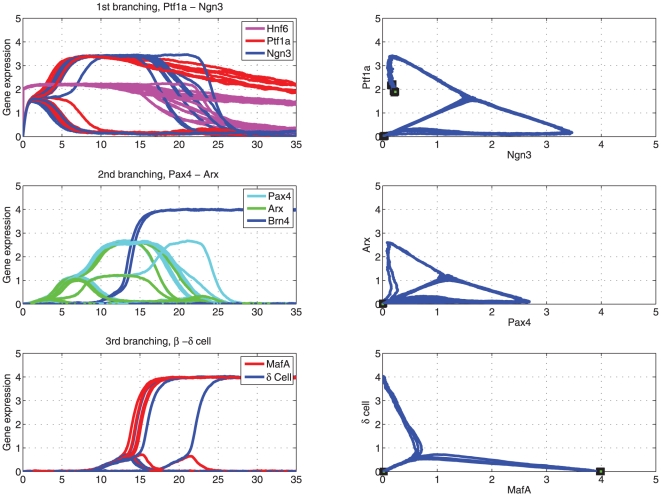
Trajectories of three pancreatic cell differentiations in the phase space. The three coordinates are the three largest components of principle components analysis (PCA) of all trajectories of pancreatic cell differentiation. We run our model in cell population and record gene expression trajectories of all cells. Then we employ PCA to analyze these data and choose three largest components for visualization.

Since development driven by the change of gene expression profiles should be represented by a trajectory in a *N*-dimensional state space (*N* = 11) of the GRN, the above separate representations in the two dimensional phase planes of switch genes do not do justice to the integrated dynamics of the entire GRN. To visualize the high-dimensional trajectories and capture the entire dynamics, we used principle components analysis (PCA) on the gene expression profiles and plotted trajectories in the phase space of the three largest principal components (Fig. 4). The PCA trajectories show the sequence of the binary decisions split the trajectories from the progenitor state to various terminal cell types. The characteristic “common trajectory” before the branching event reflects the destabilization of the respective progenitor state as previously observed for the hematopoietic system [22].

**Figure 5:**
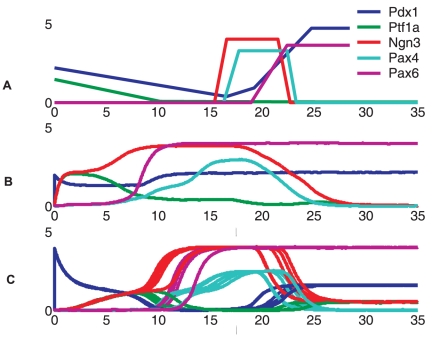
Temporal gene expression profile during pancreatic cell differentiation. In this figure different colors denote different genes. (A) Experimental observations of both gene expression levels and timing are qualitative reported in [18]. (B) Simulation results from the master model. (C) Simulation result of an alternative model with the inhibitory effects of Pdx1 upon *Ngn3 and Ptf1a.*

We also compared the predicted temporal evolution of individual genes with the experimental gene expression data during endocrine β cell development (Fig. 5). As Fig. 5B shows, the temporal profile predicted by the GRN model recapitulates qualitatively the key features, notably the counter-intuitive non-monotonical behaviour of key TFs. For instance, the model predicts the transient expression of the genes Ngn3 and Pax4 which disappear in the mature cells despite being essential fate determining factors for endocrine cells. Similarly, the transient decrease followed by the terminal increase in the case of Pdx1 and terminal increase of Pax6 is reproduced by the dynamics of the GRN. However, the detailed time course predicted still deviates from the observed time profiles, notably the sharp temporal changes of the transient appearance of Ngn3 and Pax4 (Fig. 5B). Could we construct a model to fit the experiment data better? We think that the main parts of the model are constrained by the data and cannot be changed; i.e., three gene switches and the positive feedbacks onto Pdx1 represent the core requirement. However, since Pdx1 is anti-correlated with Ngn3 and Ptf1a, it could only either inhibit the two latter genes or have no direct action upon them. As Fig. 5C shows, such behaviour can be achieved if a speculative manually wired variant GRN is allowed in which Pdx1 exerts an inhibitory effect on expression of Ngn3 and its opponent Ptf1a. While such a direct regulatory function of Pdx1 on Ngn3 and Ptf1a has not been reported, this manipulation exemplifies the possibility of dynamical models for hypothesis generation, allowing us to postulate the existence of regulatory links based on dynamical behaviour of expression. The final gene expression patterns of four distinct pancreas cell types, exocrine cell, *α* cell, *δ* cell and *β* cell, are presented in Fig. S2 Comparison with the observed gene expression patterns (Fig. 1) shows good agreement of the attractor state gene expression profiles of our model with the experimental results.

### Perturbations and reprogramming

#### Prediction of gene knock-out experiments

**Figure 6:**
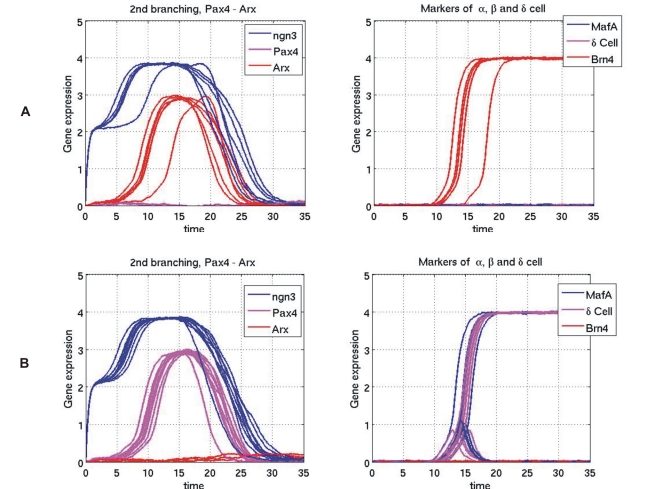
The gene expression profiles of knock out simulations. (A) The case of knocking out *Pax4*: α cell marker *Brn4* is expressed while no *MafA and δ cell* gene are expressed; (B) The case of knocking out *Arx*: *MafA and δ cell gene* are expressed while *Brn4* is not expressed.

We next evaluated whether the simple formalization as a dynamical system of the known qualitative gene regulatory relationships can predict the consequence of gene knock-out experiments [35]. Here we simulate a genetic deletion (knock-out) of a transcription factor *X_i_* by holding its expression value to be zero (*X_i_* = 0) for all time. Not surprisingly, our simulations show that cells develop into only one of the two accessible cell lineages downstream if we knock out one gene in the binary gene switch. For example, deleting the Pax4 gene results in the absence of β or δ cells and only the α cell marker Brn4 is highly expressed, as shown in Fig. 6(A). Similarly, knocking out Arx will suppress α cells while only β and δ cells develop, as shown in Fig. 6(B).

**Figure 7:**
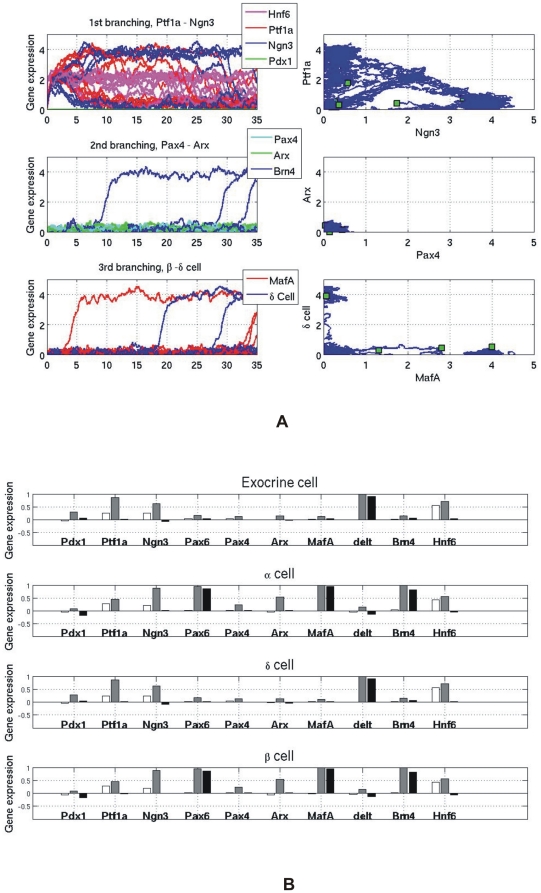
Noisy gene expression profiles after knocking out *Pdx1*. (A) Because all fully differentiated cells have a positive feedback, their marker genes can be activated by noise. Exocrine, α, β and δ cells appear, but their steady state gene expression patterns are different from the normal cells. (B) Noisy gene expression patterns of Exocrine, α, β and δ cells after knocking out Pdx1. White – Initial values; Gray – Maximum value; Black – Final value.

The predictions of consequences of gene knockouts are not always absolutely correct. For instance, in the knockout of the master gene Pdx1, there is pancreas agenesis as expected. However, a small amount of differentiated pancreas cells can be found in Pdx1 deficient mice as reported in some experiments [29], [36], [37]. This result challenged the views which consider cell types as resulting from a simple combination of TF actions during development. But in the view that cell types are attractors in a dynamic system, the high-dimensional attractor may, albeit in less stable form, still persist after deletion of a single node of a complex network [38]. To uncover the potential “hidden' attractors that may resemble that of differentiated cells, we hold Pdx1 gene expression to be zero on average but increase gene expression amplitude for the stochastic fluctuations. Simulation of the network with such high-noise dynamics then indeed reveal the presence of stable differentiated pancreas cells in the absence of Pdx1 activity, as shown in Fig. 7A. However, the terminal gene expression patterns of the four cell types differ somewhat from those of the wild-type network in the presence of Pdx1 activity. The difference is shown in Fig. 7B. For instance, different effector genes specific for the different cell types can co-exist in the same cell which suggested mal-functions of these cells. Such promiscuous marker misexpression is a common observation in cancer where the GRN architecture is altered by mutations.

#### Simulation of reprogramming with genes *Pdx1^+^*, *Ngn3* ^+^ and *MafA^+^*

Melton's laboratory recently reported that overexpression of *Pdx1^+^, Ngn3^+^ and MafA^+^* could reprogram exocrine pancreas cells to the insulin producing β cells [23]. To model such reprogramming, we describe the virus-mediated ectopic gene overexpression with temporal additional gain terms in the corresponding equations in our GRN model. Then the dynamics of the modified network is simulated as above. Various scenarios of the reprogramming experiments were computed in our model and results are compared with the experiment data.

**Figure 8:**
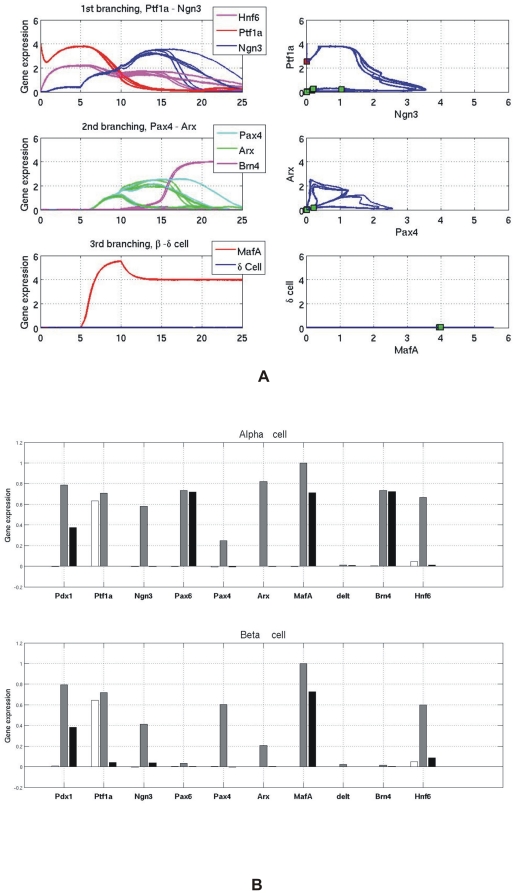
The gene expression profiles of cell reprogramming with the recipe *Pdx1, Ngn3 and MafA.* (A) Gene expression profiles of pancreatic exocrine cells being reprogrammed to β cells. Three genes, *Pdx1, Ngn3 and MafA* are over-expressed as the extra production terms in the model. Besides β cells appear, α cells also appear after reprogramming. (B) Ten gene expression patterns in reprogrammed pancreas α and β cells with the recipe: *Pdx1, Ngn3 and MafA*. White – Initial values; Gray – Maximum value; Black – Final value.

Fig. 8A presents the gene expression time profiles and trajectories in the relevant phase planes during cell reprogramming using over-expression of genes *Pdx1^+^, Ngn3^+^ and MafA^+^*. An exocrine cell starts with high expression of Ptf1a. Reprogramming is implemented by the extra production terms *for Pdx1, Ngn3 and MafA* in a certain time window. We see that the cell switches its expression pattern from a high-Ptf1a to a high-Ngn3 pattern which subsequently triggers the cell to go through the Pax4-Arx branch point to finally reach the steady state of the beta cell. It should be noted that some α cells are also produced in this process. The final gene expression patterns of reprogrammed cells are shown in Fig. 8B, which are identical to the one of normally developed α and β cells (see Fig S2).

The model also allows us to investigate how cells respond when applying these perturbations at different time sequences. Four scenarios are designed for this purpose: (A) Pdx1, Ngn3, MafA; (B) Ngn3, Pdx1, MafA; (C) MafA, Ngn3, Pdx1; and (D) MafA, Ptf1a, Ngn3. As shown in Fig. S4, optimal reprogramming is achieved by the perturbation sequences in which MafA and Ngn3 are perturbed first. This is because Ngn3 is activated early and lasts for a long time, which is good for cell reprogramming (See A and C in Fig. S4). One reason behind the differential effect of these perturbation sequences is that early MafA perturbation has a positive feedback to activate Pdx1 early accordingly, which strengthen Pdx1's activation of Pax4 and the switch to the endocrine cell lineage. Thus, varying perturbation sequences may further increase the reprogramming efficiency for which so far simultaneous perturbations has been employed.

#### Predictions of new reprogramming protocols: Pax4^+^ and Ptf1a^−^

**Figure 9:**
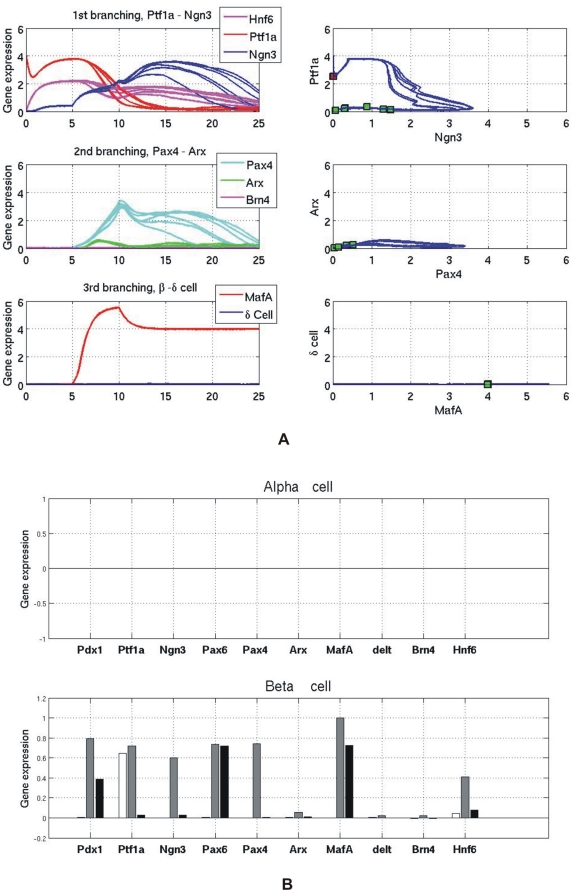
The gene expression profiles of cell reprogramming with the recipe *Pdx1, Ngn3, Pax4 and MafA.* (A) Gene expression profiles of pancreatic exocrine cells being reprogrammed to β cells. Four genes, *Pdx1, Ngn3, Pax4 and MafA* are over-expressed as the extra production terms in the model. Only β cells appear after the cell reprogramming. No α cells appear since they are repressed with the introduction of the gene *Pax4*. (B) Ten gene expression patterns in reprogrammed pancreas β cell with the recipe: *Pdx1, Ngn3, MafA and Pax4.* White – Initial values; Gray – Maximum value; Black – Final value.

According to the GRN model (Fig. 1 and 2), Pax4 is on the branching point, tilting the balance towards *β* cells. It is natural to assume that adding it to the set of TFs used by the Melton group for reprogramming *β* cells can increase the reprogramming efficiency. Fig. 9A shows that indeed the chance of *β* cell reprogramming is increased when Pax4^+^ is induced in addition to *Pdx1^+^, Ngn3^+^ and MafA^+^*. Compared with Fig. 8, there are no α cells after cell reprogramming. Because cells are prevented from becoming α cells and are channelled more towards β cells. The reprogrammed cells also exhibit the normal β cell gene expression pattern as shown in Fig. 9B (see also Fig. S2).

Model simulations also predict that Ngn3's role in the reprogramming can be enhanced by the inhibition of Ptf1a directly. It also works to inhibit Ptf1a gene (e.g. using RNAi technology) as well as to over-express Ngn3 for the purpose of reprogramming. Since Ptf1a and Ngn3 are cross-inhibitory, when Ptf1a expression level is suppressed, Ngn3 expression will increase. The inhibition of Ptf1a is modeled as an extra degradation term in the equation for a certain time window. Our further simulation (Fig. S3) shows that in addition to over-expressing genes, the inhibition of Ptf1a can in principle also result in efficient *β* cell reprogramming.

## Discussion

The purpose of this paper is to demonstrate how a gene regulatory network, built with reported interaction schemes which mostly represent causal networks, in principle governs cell type diversification and differentiation. Therefore, our dynamical model omits many connections in the regulatory gene network as well as higher level control mechanism, such as cell-cell interactions, population level quorum sensing and tissue mechanics. Thus, while our model correctly predicts the distinct gene expression patterns embodied by the various pancreas cell types, it does not predict the fraction of each cell type in the tissue which may depend on tissue homeostasis mechanisms. For the same reason, we did not seek to find the experimental values of rate coefficients of gene interactions or to fit the model to the data. Instead, cell differentiation and terminal states are quite robust (“structurally stable” in the sense of dynamical system) and is mostly determined by the topology of the gene regulatory network. Using parameter scanning we found that indeed our model is qualitatively robust in a wide range of individual parameter variations, which include the production rate ***a***, the degradation rate ***k*** and the signal weakening coefficient **η**, as shown in Fig. S1.

We also found that other functional forms for the equations, which only capture the phenomenological causal relationships between the variables rather than represent chemical reaction kinetics, yielded the same qualitative results as long as the input-output relationship obeys a sufficiently sigmoidal function. Thus, it is important to note that a high value of *n* does not indicate any assumption of molecular cooperativity in the sense of multimer reaction [39], but is used only to make the transfer functions sigmoidal which can have a multitude of reasons.

Our results show that with a minimum of knowledge of the constraints imposed by the gene network topology, pancreas cell differentiation can be explained as the transitions among different cell attractors. Specifically, the gene expression patterns of these stable steady states and the time course of the gene expression predicted by our model simulation agree with the experimental data qualitatively. Of note, the stochastic model, which captures the ubiquitously observed noisy nature of cell fate determination [33], also allows for rare spontaneous attractor transitions, explaining the presence of the few unexpected endocrine cells observed in Pdx1 knockout experiments.

In addition to reproducing observed behaviours in pancreas cell reprogramming, we also can make predictions on aspects of reprogramming that have not yet been experimentally tested. First, exocrine to β cell reprogramming should also concomitantly generate some new α cells. Although the current reprogramming protocol can switch exocrine cells to β cells, it does not prevent them from choosing other branches, including to α cells. Second, extra Pax4 induction could lead to more efficient reprogramming than the original protocol. As shown in the last section, adding *Pax4* can push more reprogrammed cells into the β cell lineage. We also found by modeling that over-expressing genes *Pdx1, Ngn3* and *MafA* combined with the suppression of *Ptf1a* would enhance the efficiency of β-cell cell reprogramming. This is important because it is often technically easier to suppress (using small molecules or RNAi) rather than over-activate genes. A mathematical model could provide the means to systematically identify the set of nodes which need to be inhibited rather than activated to achieve desired cell type transition. Also, we evaluated the influence of different perturbation sequences upon cell reprogramming. We found that with the same perturbation set (encompassing Pdx1, Ngn3 and MafA), the optimal perturbation sequence would be to perturb MafA or Pdx1 first. Playing with perturbation sequence adds a new dimension to optimize the design of the recipe for cell reprogramming.

In the coming years, we will certainly encounter more and more reprogramming experiments of different cell types. Our work shows that even with qualitative and incomplete information of interactions of the key genes for cell lineages, we can build a mathematical model to describe the cell differentiation process. After validating the crude network dynamics with the observed gene expression behaviour during cell differentiation, we can employ the model to predict the appropriate gene combination for the desired cell reprogramming. This approach builds a testable model to guide the discovery of cell reprogramming recipes instead of depending on qualitative guesswork and trial and errors, and thus, will pave the road to more efficient reprogramming protocols for regenerative medicine.

We think that this framework of modeling cell differentiation as a multi-step hierarchical branching in which intermediate potent progenitor cells are metastable states has wide validity in tissues beyond pancreas because of similarities of gene circuit motifs in many tissues: cross-inhibitory gene pairs include that control binary fate switches include Cdx2 and Oct4 in early pluripotent ES cells, and GATA6 and Nanog in the inner cell mass or GATA.1 and PU.1 in hematopoietic cells.

Future incorporation of more gene regulatory interactions and their detailed interaction properties can be explored as new network data arrive to improve the quality of predictions and extend them to other cell types. Another improvement is to incorporate the cell-cell interactions in our GRN model. Non-cell autonomous phenomena, mostly embodied by cell-cell communication, is underexplored and perhaps has evolved to control the relative proportions of cell types. We note that while our model produced correct cell types, the ratios were incorrect – perhaps because of the lack of intercellular communication – since even if the internal parameters were tuned to fit the observed relative proportions of cell types, it may not be structurally robust. The robustness of number distribution of each cell type could be an attractor in a bigger tissue level network that considers cell-cell interactions [40], [41], [42], [43].

## Methods

### Network definition - Choosing the nodes

The nodes (genes) of a GRN model that can generate the attractors representing the cell fates of interest must first contain the TF genes involved in the mutual repression circuits detailed above: *Ptf1a* and *Ngn3*; *Pax4* and *Arx*; as well as *MafA* and a putative ‘δ gene’, as discussed before. In agreement with their role in the binary switch, transgenic knockout mice of some of these regulators led to impairment of the lineages for which they act as fate-determining factor (reviewed in [26]): Ptf1a-knockout mice exhibit complete absence of the exocrine pancreas, Ngn3-knockout conversely results in complete absence of endocrine cells. Pax4 knockout led to the absence of β and δ cells with concomitant increase in α cells, whereas Arx knockout mice exhibit an increase of β/δ cells at the expense of α cells [44].

Second, in addition to these 6 genes involved in the bistable circuits, we included the following genes whose roles in pancreas development are well documented, with the respective specific rationales. *Pdx1*, the master gene for pancreas development is included because of its critical role as transcriptional activator of various pancreas development genes (see below). Knock-out of *Pdx1* results in pancreas agenesis. Another ‘upstream’ TF, *Hnf6,* was included as a key representative of a family of TFs expressed early in pancreas development but is not strictly pancreas specific. Hnf6 is expressed in the foregut prior to *Pdx1* and transactivates *Pdx1* and other genes [33]. *Hnf6* knockout mice are deficient in endocrine pancreas as well as bile duct development.

The gene *Pax6* was included because of its well-documented role in the genesis of α-cells. Pax6 knockout mice lack α cells, but the functionality of β cells and other endocrine cells is also affected [45]. The TF *Brn4* was included as a ‘downstream’ acting TF to maintain symmetry between the lineages so as to ensure the same time scale for differentiation of each the cell types (several days for each). It does not appear to play a role in binary decisions.

For many of the genes chosen for our GRN model the temporal profile of their expression levels during endocrine cell development is also fairly well established (Fig. 5A), offering a means to validate the dynamical model.

### Network definition - Choosing the connections

The network connections are directed regulatory relationships (represented by arrows between nodes, Fig. 2) and were extracted from the literature (the summary is shown in Table 1) [16], [26], [27], [28], [46], [47]. For a connection “A→B” to be qualified as a directed edge in the network, one of the following minimal criteria of evidence has to be met: (1) direct molecular evidence of binding and functional studies, i.e. A binds to the promoter of B or in the case of inhibition; A–B protein-protein interaction; (2) overexpression or knockout of A changes the expression of B accordingly; (3) binding of A to promoter of B based on ChIP data or, as a weaker criterion, presence of canonical response element for A in the promoter of B. Thus, the criteria are of heterogeneous stringency, covering a range from inferred, physical to functional interactions.

**Table 1:** Gene interactions based on references and based on proposal.

No.	Gene	Action	Gene	Direct	References
**1**	Hnf6	activate	Ngn3	direct	[34]
**2**	Hnf6	activate	Pdx1	indirect	[17]
**3**	Ngn3	inhibit	Ptf1a	indirect	[17]
**4**	Ngn3	activate	Pax6	direct	[15]
**5**	Ngn3	activate	Pax4	direct	[17]
**6**	Ngn3	activate	Arx	direct	[26]
**7**	Pax4	inhibit	Arx	direct	[15]
**8**	Pax4	activate	MafA	direct	proposal
**9**	Arx	inhibit	Pax4	Direct	[15]
**10**	MafA	activate	Pdx1	direct	[15]
**11**	Pax6	activate	Pdx1	direct	[15]
**12**	Pdx1	activate	Pax4	direct	[17]
**13**	Pdx1	activate	Arx	direct	proposal
**14**	Pdx1	activate	MafA	direct	proposal
**15**	MafA	self- activate	—	direct	proposal
**16**	Arx	activate	Brn4	direct	[26]
**17**	Brn4	self- activate	—	direct	proposal

The above criteria are minimally necessary but not sufficient for inclusion in the model so that not all known documented interactions in published papers that satisfy the criteria are included. This resulted in the network shown in main text Fig. 2. Since very little is known about how the individual bistable circuits interact in addition to the connections mentioned above the following interactions were added as justified below:

Hnf6, which transactivates not only Pdx1 but also the two members of the first bistable switch (Ptf1a and Ngn3) [19], may be responsible for initiating the first decision point. Pdx1 transactivates the “downstream” TFs, MafA and Pax4 [26], but interestingly, does not appear to affect the first decision circuit, Ptf1a<--> Ngn3. Although Pdx1 seems to be at the top of the hierarchy in development of the pancreas parenchyma and to be critical for exocrine and endocrine pancreas, almost no gene regulatory findings are known that would explicitly and obviously explain how the cascade of cell fate decision circuits is initiated by Pdx1. Thus, it appears that it is Hfn6 which triggers the cascade of subsequent binary decisions. However, Pdx1 is, based on promoter binding site analysis, subjected to autoregulation [36] and regulation by MafA, Pax4 and Pax6 [26]. These feedback interactions were included in view of the well known non-monotonical time course of Pdx1 during pancreas development (Fig. 5A) and these connections are incorporated in the model.

Ngn3, which is the master regulator for the endocrine pancreas, was modeled as activator of the targets Pax4, Arx, Pax6 and MafA based on promoter binding sites and functional evidence [48] since these regulatory relationship may account for activation of the downstream decision circuits.

Since little information is available for fate determining TF for the δ cells, for modeling purposes and for maintaining symmetry, we use a place-holder for the δ-cell determining TF, called “δ factor”. As we assume that the cross-inhibition genes stand at the branching point of each cell differentiation, a pair of opposing TFs, MafA <- ->δ factor, is proposed to govern the determination of β vs. δ cells respectively.

### Simplifications and deviation from data

Although in this subnetwork not all genes known to play a role in pancreas development are included, in expanding the above selection some genes with similar functions are lumped into one variable. For instance, NeuronD and Isl1 were grouped with Ngn3. Exocrine cell marker Mist1 is lumped with Ptf1a.

In addition to the above genes we also defined a network node, the hypothetical variable “*maturation*” to capture the functional role of feedback signals emanating from the maturing tissue, such as possible regulatory signals from increasing cell density and the presence of differentiated cells. i.e., the fully differentiated cells would send inhibition signal to triggering gene Hnf6 via “maturation” node. This is necessary since unlike in other cell differentiation systems studied, such as hematopoietic stem cells, embryonic stem cells or glial cells, where intrinsic robustness and context independent cell type diversification in vitro provide a global driving force, pancreas is no strong evidence for such developmental autonomy and hence, a maturation factor is necessary as an extrinsic reference of time progression that drives and constrains the dynamics. Here Ptf1a, MafA, δ-gene, and Brn4 are marker genes of fully-differentiated cells, which are expressed stably in these cells. Their expressions represent the maturation of cells and send signals to “maturation”[49], [50], [51], [52]. Hnf6 is the trigger point of the differentiation and no gene acts on it. So it is the right gene to receive the feedback after the cell maturation.

The characteristic of our approach is to investigate the mechanism of cell lineage determination with an incomplete network. Here the backbones of our GRN are three cross-inhibition gene switches and the links between them. If any of them is removed, cell differentiation will collapse in the model. All other genes and connections are included to make the gene expression profiles replicate the observations. In this sense, the ‘maturity’ and its connections are not essential for cell differentiation. It is not necessary because it is only used to simulate the effects of the surrounding cell population.

We also systematically implemented self-activation for fate-determining TFs. Such positive feedback has been found in many TFs involved in fate determination including for MafA and Pax6 in the case of pancreas [16], [26] and are well described in other systems [3], [29]. They contribute to stabilizing the progenitor state as well as to separating and stabilising the distinct lineages [23]. However, the lack of autoregulation in the upper switches is due to the fact that some cross-inhibition genes are only transiently expressed during pancreas development. Autoregulation in the gene switch can keep it on even after upstream signals are gone. According to experiment data, both Ngn3 and Pax4 are only transiently expressed. Therefore, we cannot have autoregulation for the first two switches.

Conversely, known negative feedback loops where a TF directly inhibits its own synthesis/activation, as in the case of Ngn3 and Pax4, were omitted since their incorporation made no difference for the dynamics in the parameter space examined. The detailed list of auto-regulation for each gene can be found in Table 1.

In order to generate a model, we used the above, well-studied general principles of a network of cross- and auto-regulation in which the rate of expression change of the downstream target is a sigmoidal function of the upstream regulators. For the functional form of the latter, Hill functions with uniform exponents *n* (n = 4) were used. It should be noted that using Hill functions does not imply cooperativity of protein binding in this coarse-grained network model. The real event of regulated protein expression is far too complex, involving multiple cellular processes, such as chromatin confirmation changes, transcription initiation and transcript elongation, nuclear export and splicing and hundreds of steps in translation and formation of active proteins so that a direct mapping of individual molecular events to observable kinetics of protein concentrations in cell populations is not warranted [53], [54], [39]. Many conditions, including stochastic focusing, non-Michaelis-Menten and fractal kinetics, non-ideal chemistry, circuit structure, etc. can give rise to sigmoidal kinetics [55], [56]. Moreover, the typical dynamics of networks with attractors can be obtained with sigmoidal functions of forms other than Hill functions. Finally, each TF also is subjected to non-regulated first order degradation.

To show the dynamic bifurcations arise from the topology of the gene regulatory network rather than from the arti-fact of some special parameters, we ran the simulations with varying parameter values, covering the range of two order of the scale [57] (Fig. S1). Any set of parameters which produce the four expected cell types were plotted as solid line in the left panel of Fig. S1. It demonstrates that large number of parameters could lead to the proper bifurcations in our model.

### The rate equations for the GRN of pancreas cell development

Below are the ordinary differential equations for the network of regulatory influences shown in Fig. 2. 
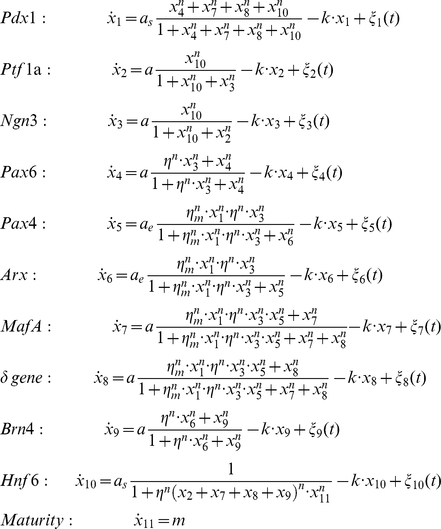



To keep the model simple and minimal, we kept the number of parameters which characterize the interactions to the minimum. Here variables x_1_ ∼x_10_ represent the expression level of 10 key genes in pancreas cell differentiation. In addition to the above genes, we also defined x_11_ as a hypothetical variable “Maturity”, which, as explained above, is not a parameter to trigger cell differentiation but a variable to captures the functional role of feedback signals emanating from the fully differentiated cells. It is worth to note that x_11_ is not a bifurcation parameter. Cell differentiation can happen without the “Maturity” factor x_11_, which only influences how fast Hnf6 degrades after mature cells appear. Each equation has three terms to capture the effects upon production from the upstream TFs, the linear degradation and stochastic gene expression. Transcription and translation (and posttranslational activation) –processes that are independent of the actual GRN architecture were lumped together as the rate of change of the gene expression *x*_i_ of each TF since they operate at a time scale (hours) much smaller than the differentiation (week). The production rate of expression change of the downstream target is a sigmoidal function of the upstream regulators. Hill functions with uniform exponents *n* (*n* = 4) were used which, as explained earlier, do not imply cooperativity. To demonstrate how the network architecture and qualitative interactions can generate the observed cell fate dynamics, all rate coefficients have the values near one and do not depend on fine tuning or exact experimental data fitting. We started with the same parameters for all equations and then minimally adjusted them to allow for multi-stable dynamics and to agree with the gene expression temporal profiles qualitatively. The values of production rate *a* and degradation rate *k* are chosen only such that the bifurcations can happen. Pdx1 and Hnf6's production rates are smaller while those of Pax4 and Arx are larger than the ones of other genes solely to keep the steady states around similar values. The complete list of parameter values are listed in Table 2.

**Table 2:** Parameters values.

Parameters	Value	Notes
**production rate**		
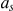	2.2	For Pdx1 and Hnf6
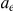	6	For Pax4 and Arx
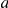	4	All other genes
**Degradation rate** 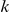	1	All genes
**weakening coefficient**		
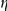	0.25	Single input
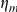	0.125	Multiple inputs
**Hill coefficient** 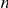	4	
**Maturity rate** 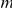	0.01	

Since it usually takes several days to weeks for the mouse pancreatic cells to fully differentiate, these node-intrinsic molecular processes that take place within hours may not play a role in global network dynamics that governs the cell differentiation. Instead, typical time scale of macroscopic differentiation of multiple days suggests that some delays between subsequent cell differentiation steps need to be accounted for. Instead of introducing time delay explicitly which complicates the model, we simply, where biologically justified, use weak coupling between the binary decision circuits, which is implemented by coefficient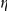. The multiple input coupling coefficients 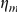 is smaller than the single input one since gene input strengths need to be normalized to the same scale.

The deterministic parts of equations describe the mean field values of the gene expression of the development network and can not exploit bifurcations to diversify cell fates. The fluctuations of gene expressions due to intrinsic and extrinsic noise are essential for the cell fate differentiation at bifurcation points (see main text). The white noise term 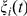 is added for each equation with Gaussian distribution assumed. The auto-correlations of noise are given:
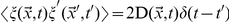



Where 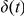 is the Dirac delta function and the diffusion matrix 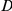 is defined by 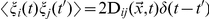. The average is carried out with the Gaussian distribution for the noise. Here diffusion matrix 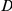 is assumed to be independent of coordinate x. In the current model, the differentiation process is quite robust because three subsequent bifurcations are not sensitive to noise level 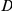. However, noise (magnitude of D) cannot be bigger than certain value (0.25 in our model). Otherwise cell attractors are no longer stable and cell types can spontaneously switch to each other during the normal development process.

The noise is implemented as discretized Brownian motion. The stochastic ODEs are solved by Euler-Maruyama method which was programmed by the author in Matlab [58].

Since cell differentiation happens at the cell population level and our gene network model only represents one single cell, the simulation is carried out in a small ensemble of cells with the same initial conditions. When these cells differentiated into different cell types of exocrine and β, δ, α endocrine cell types, their gene expression patterns and profiles are recorded separately.

## Supporting Information

Figure S1 Robustness of parameters values. To show the dynamic bifurcations arise from the topology of the gene regulatory network rather than from the artifact of some special parameters, we ran the simulations with varying parameter values, covering the range of two order of the scale [52]. Any set of parameters which produce the four expected cell types were plotted as solid line in the left panel of Fig. S1. It demonstrates that large number of parameters could lead to the proper bifurcations in our model.
(0.82 MB TIF)

Figure S2 Gene expression patterns in the four distinct pancreas cell types. Gene expression patterns in the four distinct pancreas cell types as attractors of GRN. White-initial values; gray-Maximum values; black-final values.
(1.64 MB TIF)

Figure S3 The gene expression profiles of cell reprogramming with recipe of overexpressing Pdx1, Ngn3, Pax4, MafA and inhibiting Ptf1a. This cell reprogramming scheme is the optimum to reprogram exocrine cells to beta cells.
(1.99 MB TIF)

Figure S4 The gene expression profiles of cell reprogramming with different perturbation sequence of Pdx1, Ngn3, MafA. A) Pdx1, Ngn3, MafA; B) Ngn3, Pdx1, MafA; C) MafA, Ngn3, Pdx1; D) MafA, Ptf1a, Ngn3.
(1.51 MB EPS)
