# Supplementary figures and images for: Predicting Pancreas Cell Fate Decisions and Reprogramming with a Hierarchical Multi-Attractor Model
Source: PLoS One. 2011 Mar 14;6(3):e14752. doi: 10.1371/journal.pone.0014752 (PMC3056652; doi:10.1371/journal.pone.0014752)

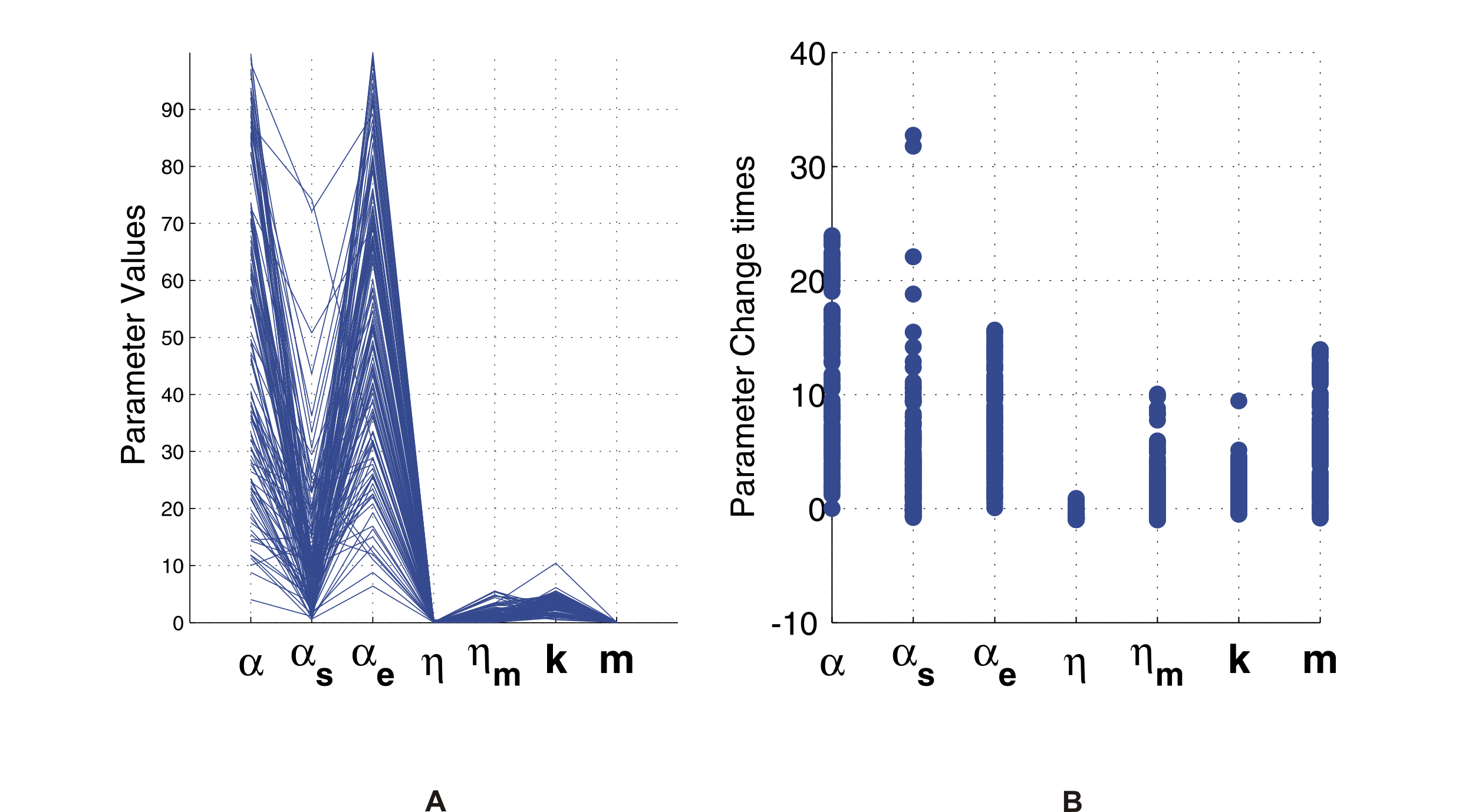

Supplement: Figure S1 — Robustness of parameters values. To show the dynamic bifurcations arise from the topology of the gene regulatory network rather than from the artifact of some special parameters, we ran the simulations with varying parameter values, covering the range of two order of the scale [52]. Any set of parameters which produce the four expected cell types were plotted as solid line in the left panel of Fig. S1. It demonstrates that large number of parameters could lead to the proper bifurcations in our model. (0.82 MB TIF) [file pone.0014752.s001.tif]

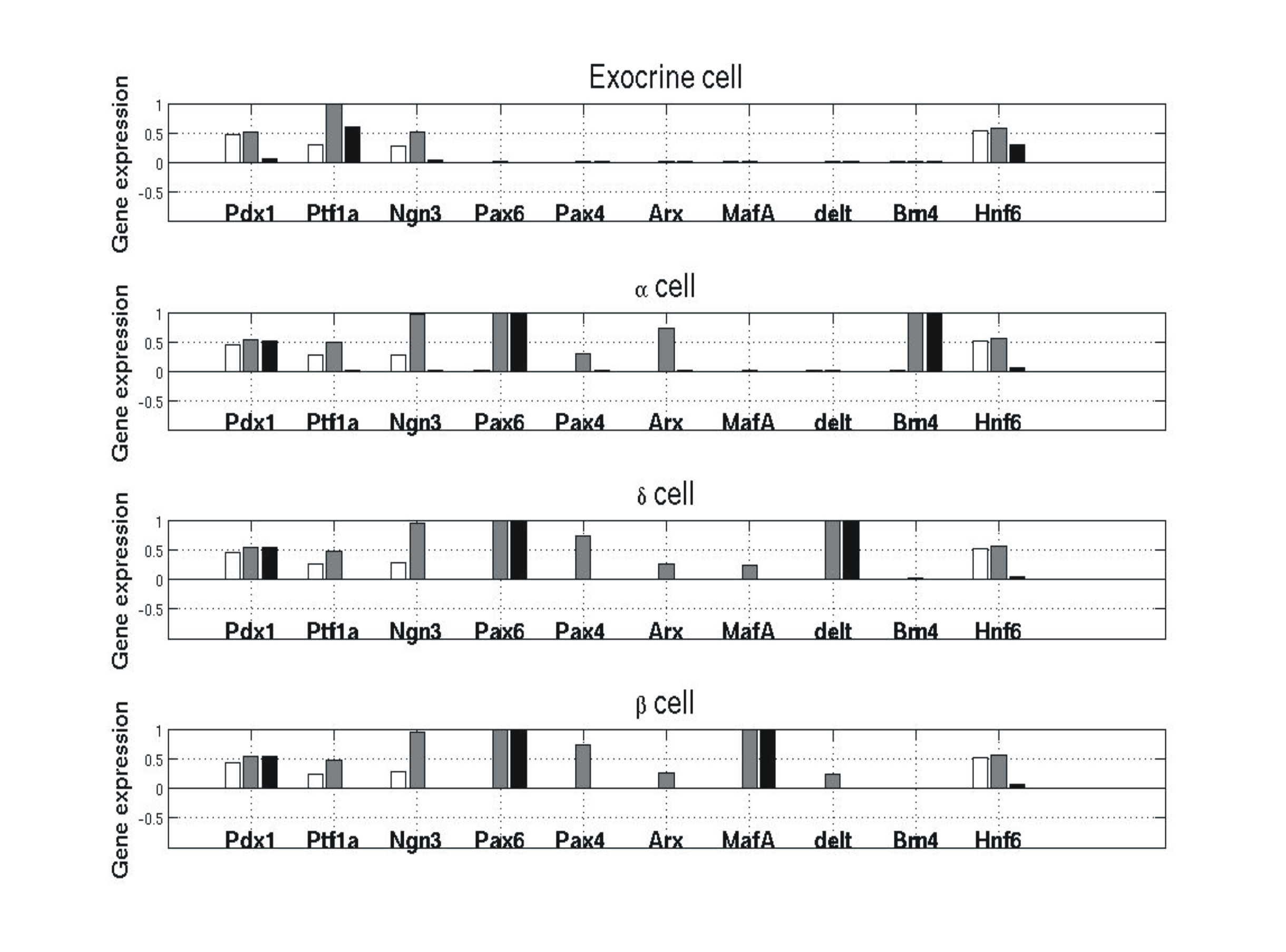

Supplement: Figure S2 — Gene expression patterns in the four distinct pancreas cell types. Gene expression patterns in the four distinct pancreas cell types as attractors of GRN. White-initial values; gray-Maximum values; black-final values. (1.64 MB TIF) [file pone.0014752.s002.tif]

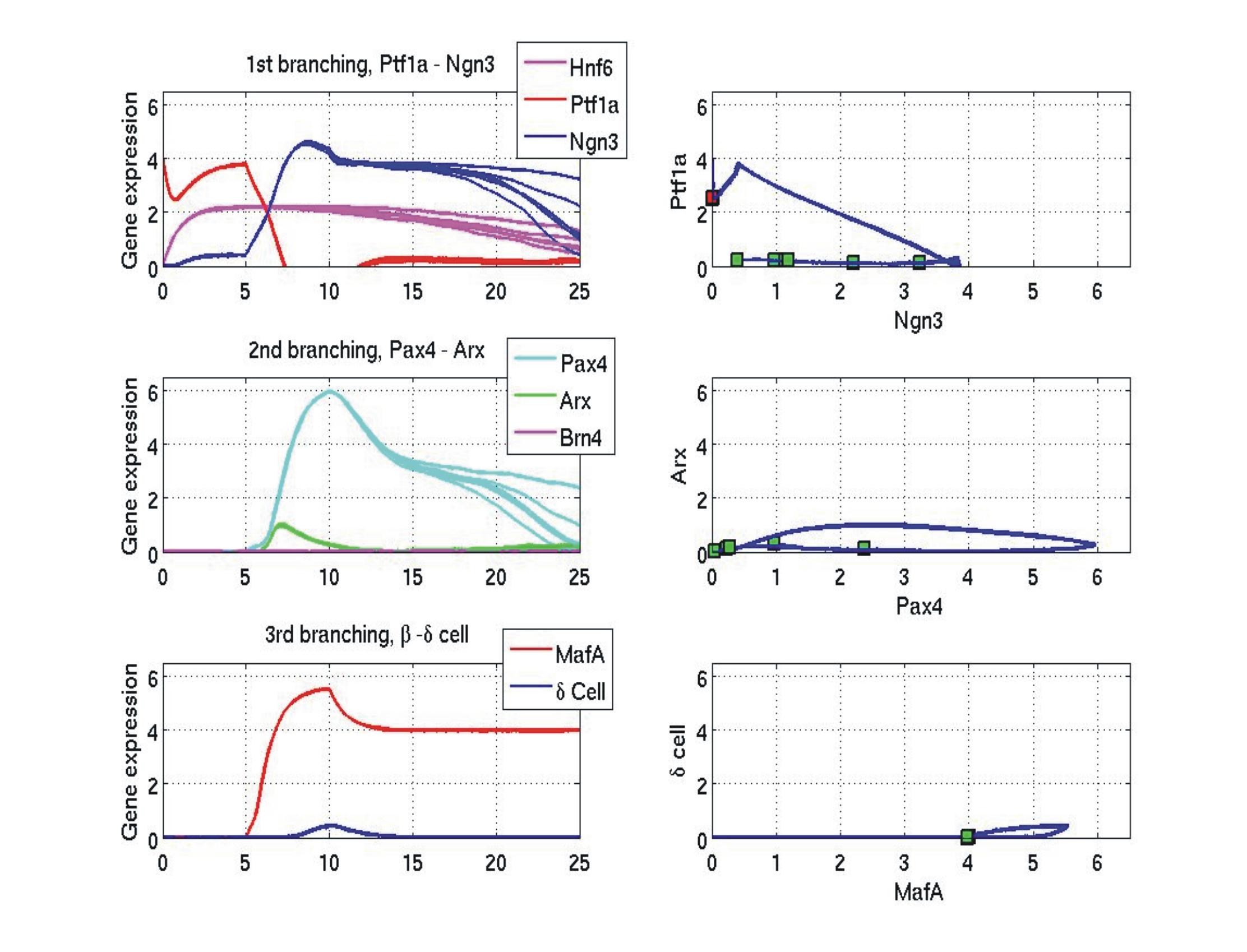

Supplement: Figure S3 — The gene expression profiles of cell reprogramming with recipe of overexpressing Pdx1, Ngn3, Pax4, MafA and inhibiting Ptf1a. This cell reprogramming scheme is the optimum to reprogram exocrine cells to beta cells. (1.99 MB TIF) [file pone.0014752.s003.tif]
